# Nurse-led rehabilitation after total hip or knee arthroplasty: a systematic review and meta-analysis of functional recovery, self-efficacy, and quality of life

**DOI:** 10.3389/fmed.2026.1911533

**Published:** 2026-10-05

**Authors:** Ying Xu, Yan Chen, Yuan Yin, Mengxia Tang, Qin Luo

**Affiliations:** Department of Rehabilitation, Taixing People’s Hospital, Taixing, Jiangsu, China

**Keywords:** nurse-led rehabilitation, quality of life, self-efficacy, total hip arthroplasty, total knee arthroplasty

## Abstract

**Background:**

Nurse-led rehabilitation is increasingly embedded in postdischarge care after total hip or knee arthroplasty, but its effect on patient-reported recovery has not been quantified through meta-analysis.

**Objective:**

The aim of this study was to evaluate the effects of nurse-led rehabilitation versus usual care on functional recovery, self-efficacy, and quality of life after total hip or knee arthroplasty.

**Methods:**

PubMed, Scopus, Web of Science, and ClinicalTrials.gov were searched (2010–2026; English only) for randomized controlled trials comparing nurse-led rehabilitation with usual care. Standardized mean differences (Hedges *g*) were pooled using random-effects models with Hartung–Knapp confidence intervals. Heterogeneity was assessed with *I*^2^ and prediction intervals, robustness with leave-one-out and subgroup analyses, and certainty with Grading of Recommendations Assessment, Development and Evaluation (GRADE).

**Results:**

Eleven trials (1,107 participants) were included. Nurse-led rehabilitation improved functional recovery (10 trials; *g* = 1.11, 95% confidence interval 0.42–1.81; *I*^2^ = 94.5%), self-efficacy (five trials; *g* = 1.10, 0.00–2.20; *I*^2^ = 93.8%), and quality of life (four trials; *g* = 0.77, 0.08–1.46; *I*^2^ = 81.7%). The certainty of evidence (GRADE) was rated low for all three primary outcomes. Secondary outcomes were directionally favorable but imprecise. The functional recovery effect was robust to leave-one-out analysis, whereas self-efficacy was statistically fragile. Quality of life showed a homogeneous benefit after knee replacement (*g* = 1.12, *I*^2^ = 0%) but not hip (between-subgroup *p* = 0.032), and functional recovery differed by delivery model (*p* = 0.012, largest for digital). Heterogeneity was substantial throughout, all prediction intervals crossed the null, and funnel plot asymmetry suggested possible small-study effects.

**Conclusions:**

Nurse-led rehabilitation after hip or knee arthroplasty was associated with improved function, self-efficacy, and quality of life, consistently favoring nurse-led care in direction but with evidence of low certainty, substantial between-study heterogeneity, prediction intervals that crossed the null, and possible small-study effects. Therefore, the magnitude of benefit remains uncertain. Adequately powered, outcome-blinded trials are needed.

**Systematic Review Registration:**

https://www.crd.york.ac.uk/PROSPERO/view/CRD420261425216, PROSPERO CRD420261425216.

## Introduction

Osteoarthritis is the most common form of arthritis and a leading global cause of chronic pain and disability. The Global Burden of Disease Study 2021 estimated that 595 million people were living with osteoarthritis in 2020—7.6% of the world's population and a 132% increase in total cases since 1990—with the knee being the most frequently affected joint (1). Driven by population aging and rising obesity, cases are projected to rise by approximately 75% for the knee and 79% for the hip by 2050, placing escalating demand on health systems for definitive treatment (1). For end-stage hip and knee osteoarthritis, total joint arthroplasty remains the most effective intervention for relieving pain and restoring function and is among the highest-volume elective procedures performed worldwide (2).

In spite of generally favorable surgical outcomes, a substantial minority of patients do not achieve the recovery they expect. A contemporary systematic review reported that approximately 10% of patients remain dissatisfied after total knee arthroplasty, with persistent pain, stiffness, and unmet expectations among the leading drivers, and with psychological factors such as anxiety, depression, and pain catastrophizing increasingly recognized as contributors (3). Because much of the recovery trajectory unfolds after discharge, the postoperative rehabilitation period is a critical determinant of long-term function and quality of life (4).

This challenge has been amplified by changes in perioperative care. The widespread adoption of enhanced recovery after surgery (ERAS) pathways has substantially shortened hospital length of stay and reduced complications (5), shifting the burden of rehabilitation from the inpatient setting to the patient's home. Nurses are central to these pathways, spanning preoperative education, discharge planning, and postdischarge follow-up (6). Adherence to standardized care processes—a recognized determinant of care quality—is itself shaped by modifiable, improvable factors (7). Yet home-based rehabilitation depends heavily on patient self-management, and adherence to prescribed home-exercise programs is frequently poor; non-adherence is consistently associated with lower self-efficacy, inadequate guidance and feedback, and limited motivational support (8).

The growing reliance on postdischarge self-management also reflects a broader transformation in how contemporary health systems organize care. Across many countries, reforms driven by population aging, workforce shortages, and the shift toward community-based and integrated services have progressively expanded the clinical and organizational responsibilities of health professionals, redistributing tasks across multidisciplinary teams to support continuity of care (30, 31). Within this evolution, rehabilitation after major surgery is increasingly conceived as an interdisciplinary, patient-centered pathway in which surgeons, rehabilitation physicians, physiotherapists, occupational therapists, and nurses share responsibility for the transition from hospital to home. Nurses occupy a distinctive position within these collaborative models: They typically have the most sustained contact with patients across the perioperative trajectory and are therefore well placed to coordinate education, monitor recovery, and support self-management between formal therapy contacts. Nurse-led rehabilitation should accordingly be understood not as a replacement for physiotherapy-led care but as the nursing contribution embedded within multidisciplinary rehabilitation pathways.

Self-efficacy—a patient's confidence in their ability to perform recovery-related behaviors—is an established and modifiable predictor of functional outcome after joint arthroplasty (9). Interventions that strengthen self-efficacy, deliver structured education, and support self-management may therefore improve not only adherence but also patient-reported function and quality of life. Nurse-led models of care are well suited to provide this support, and structured nurse-led frameworks have been advanced across surgical and orthopedic populations, including after hip fracture (10). In parallel, the rapid growth of digital and telerehabilitation has created new channels for delivering nurse-led education and remote monitoring, with meta-analyses indicating that technology-assisted and mobile application-based rehabilitation can improve pain, function, and exercise adherence after lower-limb arthroplasty (11–13).

Despite this, the specific contribution of nurse-led rehabilitation as distinct from physiotherapy-led or technology-only interventions after total hip or knee arthroplasty has not been quantified through meta-analysis. Existing reviews have focused on telerehabilitation modality (11–13) or on ERAS length-of-stay outcomes (5) rather than on the nurse-led delivery model and its effect on patient-centered endpoints such as functional recovery, self-efficacy, and quality of life. The heterogeneity of delivery formats (in-person, telephone, digital, and multicomponent) and of outcome measures across trials further complicates interpretation, and no synthesis to date has formally examined the robustness and certainty of the evidence. We therefore conducted a systematic review and meta-analysis of randomized controlled trials (RCTs) to evaluate the effects of nurse-led rehabilitation versus usual care on functional recovery, self-efficacy, and quality of life after total hip or knee arthroplasty, and to assess the consistency, robustness, and certainty of the available evidence.

## Methods

### Protocol and registration

The present systematic review and meta-analysis were conducted and reported in accordance with the Preferred Reporting Items for Systematic Reviews and Meta-Analyses (PRISMA) 2020 statement (14). The protocol was registered prospectively with PROSPERO (CRD420261425216).

### Eligibility criteria

Studies were selected using the PICOS framework. We included (Population) adults undergoing primary total hip or total knee arthroplasty for osteoarthritis; (Intervention) nurse-led rehabilitation programs, defined as structured postoperative rehabilitation, education, or self-management support designed and primarily delivered by nurses (i.e., a registered nurse or nurse-led team was responsible for designing and delivering the core rehabilitative content, comprising at least one structured patient education, guided exercise instruction, self-management or self-efficacy support, symptom monitoring, or coordinated follow-up; this definition distinguishes nurse-led programs from physiotherapist-led or technology-only interventions in which nurses had no directive role), irrespective of delivery format (in-person, telephone, digital, or multicomponent); (Comparator) usual care, routine care, or conventional discharge education; (Outcomes) at least one of functional recovery, self-efficacy, or health-related quality of life as a primary outcome, or pain, psychological distress (anxiety/depression), or exercise adherence as a secondary outcome; and (Study design) RCTs. We excluded non-randomized studies, conference abstracts without extractable data, studies of revision arthroplasty or arthroplasty for indications other than osteoarthritis, and interventions not led by nursing staff. Only studies published in English were included.

### Information sources and search strategy

We systematically searched the electronic databases of PubMed, Scopus, Web of Science, and ClinicalTrials.gov for studies published from 2010 to 2026. The search combined controlled vocabulary and free-text terms for three concept blocks: “arthroplasty” (e.g., arthroplasty, joint replacement, hip replacement, knee replacement), “nurse-led care” (e.g., nurse-led, nursing intervention, nurse navigator, rehabilitation nursing), and “rehabilitation/recovery” (e.g., rehabilitation, self-management, education, education, telerehabilitation). Reference lists of included studies and relevant reviews were hand-searched to identify additional eligible trials.

### Study selection and data extraction

Records were deduplicated and screened by two reviewers independently at title/abstract and full-text stages, with disagreements resolved by discussion or by a third reviewer. From each included trial, two reviewers independently extracted study characteristics (author, year, country, joint and procedure, sample size per arm, intervention delivery model, comparator, and outcome instruments) and outcome data (postintervention means, standard deviations, and sample sizes per arm, or adjusted between-group estimates where these were the only data reported). Where standard deviations were not reported directly, they were derived from standard errors, 95% confidence intervals, or other reported statistics according to the methods described in the Cochrane Handbook for Systematic Reviews of Interventions (15). Trials whose standard deviations were reconstructed in this way were retained in the main analysis but examined in sensitivity analyses (see in the following). One trial that reported only covariate-adjusted between-group differences for a combined hip-and-knee cohort was not amenable to standardized pooling and was therefore summarized narratively rather than entered into the pooled estimates.

### Risk of bias assessment

Two reviewers independently assessed each trial using the Cochrane Risk of Bias 2 (RoB 2) tool (16) across its five domains: bias arising from the randomization process (D1), deviations from intended interventions (D2), missing outcome data (D3), measurement of the outcome (D4), and selection of the reported result (D5). Each domain and the overall judgment were rated as “low risk,” “some concerns,” or “high risk,” with disagreements resolved by consensus. Domain-level and study-level judgments are presented as a weighted summary plot and a traffic-light plot, respectively.

### Effect measures and data synthesis

Because outcomes were measured with heterogeneous instruments, treatment effects were expressed as the standardized mean difference using Hedges *g* with the small-sample bias correction; positive values indicate a benefit favoring nurse-led rehabilitation. Effects were pooled using a random-effects model with restricted maximum likelihood (REML) estimation of the between-study variance (*τ*^2^) and the Hartung–Knapp–Sidik–Jonkman (HKSJ) adjustment to the confidence interval (CI), which provides more conservative and better-calibrated intervals when the number of trials is small (15). For each outcome, we report the pooled *g* with its 95% confidence interval and a 95% prediction interval, the latter describing the expected range of true effects in a future setting. All tests were two-sided with significance set at *p* < 0.05.

### Assessment of heterogeneity

Between-study heterogeneity was quantified using the *I*^2^ statistic, *τ*^2^, and Cochran's *Q* test, and interpreted alongside the prediction interval (15). *I*^2^ values were interpreted as approximate thresholds of low (≤25%), moderate (≤50%), substantial (≤75%), and considerable (>75%) heterogeneity, while recognizing the imprecision of *I*^2^ when few trials are pooled.

### Subgroup and sensitivity analyses

Prespecified subgroup analyses examined effects by joint (hip vs. knee), with between-subgroup differences tested using the random-effects *Q* statistic; an exploratory subgroup analysis by delivery model was also conducted. To assess robustness, we performed the following: (i) leave-one-out analyses, recomputing each pooled estimate with one trial omitted in turn; (ii) a risk-of-bias restriction, excluding the trial at high overall risk of bias; and (iii) measurement restrictions, limiting the self-efficacy analysis to validated scales and the quality-of-life analysis to standard health-related quality-of-life instruments. Influence diagnostics were visualized with Baujat plots. Meta-regression on trial-level moderators (e.g., intervention duration, intensity, length of follow-up, or geographic setting) was considered but not undertaken: No outcome was informed by more than 10 trials, and with approximately 10 studies recommended per covariate modeled, such models would be prone to overfitting and spuriously precise estimates (15).

### Small-study effects and publication bias

For outcomes informed by at least 10 trials, small-study effects were examined using a contour-enhanced funnel plot, with funnel asymmetry assessed by Egger's regression test; a cumulative meta-analysis ordered by year of publication was used to examine the temporal stability of the pooled estimate. Funnel-plot and formal tests of asymmetry were not performed for outcomes with fewer than 10 trials, as these tests are underpowered and unreliable in that setting (15).

### Certainty of evidence

The certainty of evidence for each primary outcome was rated using the Grading of Recommendations Assessment, Development and Evaluation (GRADE) approach (17) across the domains of risk of bias, inconsistency, indirectness, imprecision, and publication bias, and summarized as high, moderate, low, or very low. Findings are presented in a GRADE summary-of-findings table.

### Software

All analyses were performed in R (version 4.5.1; R Foundation for Statistical Computing, Vienna, Austria) using the *meta* and *metafor* packages.

## Results

### Included studies and participants

Eleven RCTs met the prespecified inclusion criteria, collectively enrolling 1,107 participants who were randomized to either a nurse-led rehabilitation intervention or a usual-care comparator. The study selection process is summarized in the PRISMA 2020 flow diagram (Figure 1).

**Figure 1:**
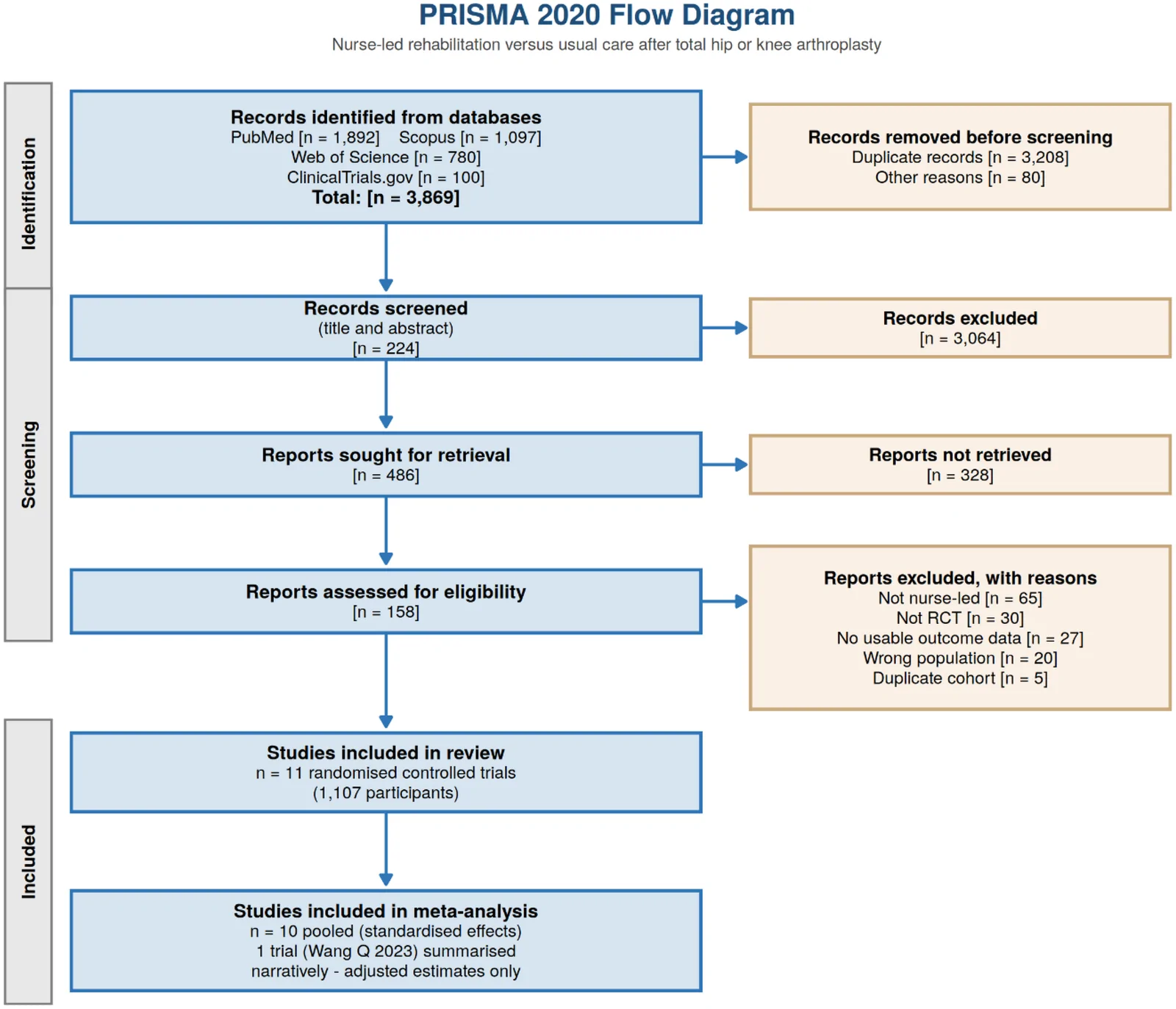
PRISMA 2020 flow diagram for the systematic review of nurse-led rehabilitation versus usual care after total hip or knee arthroplasty.

#### Geographic distribution and surgery type

Trials were conducted across five regions: East Asia (China and Taiwan, five trials), Europe (Denmark, two trials), Türkiye (three trials), South Korea (one trial), and one binational China–Australia collaboration. With respect to surgical indication, six trials recruited patients undergoing total knee arthroplasty, four trials recruited patients undergoing total hip arthroplasty, and one trial (24) enrolled a mixed cohort of hip and knee arthroplasty patients.

#### Intervention delivery modalities

The nurse-led interventions varied in their mode of delivery: four trials employed digital platforms, three implemented multicomponent programs combining education and exercise, two provided in-person education sessions, and two used structured telephone follow-up. Study-level characteristics for all included trials are summarized in Table 1.

**Table 1:** Characteristics of the included randomized controlled trials.

Study	Country	Joint	Procedure	*n* (Int/Ctrl)	Delivery model	Primary outcomes
Park and Jeong (18)	South Korea	Knee	TKA	29/28	Multicomponent	Function, wellbeing
Lin et al. (19)	Taiwan	Knee	TKR	100/100	In-person	Function, QoL
Szöts et al. (20)	Denmark	Knee	TKA	54/54	Telephone	Function, SE, QoL
Güllü and Tosun (21)	Turkey	Knee	TKA	35/35	Multicomponent	Function, SE
Boztaş et al. (22)	Turkey	Knee	TKA	33/34	Digital	SE, QoL
Biricik et al. (23)	Turkey	Knee	TKR	22/22	Digital	Function, QoL
Wang et al. (24)	China/Australia	Hip + Knee	THA + TKA	43/43	Digital	SE, function, QoL
Meng et al. (25)	China	Hip	THA	76/74	Multicomponent	SE, function, QoL
Wei et al. (26)	China	Knee	TKA	45/45	Digital	Function
Huang et al. (27)	Taiwan	Hip	THR	54/54	In-person	SE, function, QoL
Hørdam et al. (28)	Denmark	Hip	THR	59/68	Telephone	QoL/function

Int, intervention; Ctrl, control; TKA/TKR, total knee arthroplasty/replacement; THA/THR, total hip arthroplasty/replacement; SE, self-efficacy; QoL, quality of life.

One trial (24) reported covariate-adjusted mean differences for a pooled hip-and-knee cohort rather than raw arm-level means; it was therefore summarized narratively and not combined in the pooled standardized effects, but its direction of effect was concordant across all domains.

### Risk of bias

No trial was rated at low risk of bias overall. Ten trials raised some concerns, and one (28) was at high risk of bias, driven by missing outcome data. The pervasive limitation was outcome measurement: Because endpoints were patient-reported and the nurse-led intervention could not be blinded, the great majority of the weighted evidence carried some concerns for detection bias. Deviations from intended interventions raised some concerns in every trial. Domain-level weighted summary and study-level traffic-light plot are provided in the Supplementary Figures S1, S2.

### Primary outcomes

#### Functional recovery

Pooling 10 trials (*n* = 1,021), nurse-led rehabilitation produced a large improvement in physical function relative to usual care (*g* = 1.11, 95% CI 0.42–1.81; *I*^2^ = 94.5%, *τ*^2^ = 0.89). Between-trial heterogeneity was substantial, and the 95% prediction interval was wide and crossed the null (−1.13 to 3.36), indicating that the effect in an individual future setting could range from modest harm to a very large benefit (Table 2, Figure 2). The pooled point estimate was nonetheless the most robust in the review: In leave-one-out analysis, the confidence interval remained entirely above zero regardless of which trial was removed.

**Table 2:** Random-effects pooled effects for primary and secondary outcomes (REML + Hartung–Knapp, Hedges *g*).

Outcome	*k*	*g*	95% CI	95% PI	*I*^2^ (%)	*τ* ^2^
Functional recovery	10	1.11	0.42 to 1.81	−1.13 to 3.36	94.5	0.890
Self-efficacy	5	1.10	0.00 to 2.20	−1.52 to 3.71	93.8	0.729
Quality of life	4	0.77	0.08 to 1.46	−0.65 to 2.20	81.7	0.152
Pain (secondary)	3	0.35	−0.91 to 1.60	−1.80 to 2.49	68.5	0.169
Anxiety/depression (secondary)	3	0.78	−0.17 to 1.73	−0.96 to 2.52	74.3	0.111
Exercise adherence (secondary)	2	2.26	−0.36 to 4.87	−1.14 to 5.65	33.8	0.029

*k*, number of trials; *g*, pooled Hedges *g*; CI, confidence interval; PI, prediction interval; *I*^2^ and *τ*^2^, heterogeneity statistics. Positive *g* favors nurse-led care.

**Figure 2:**
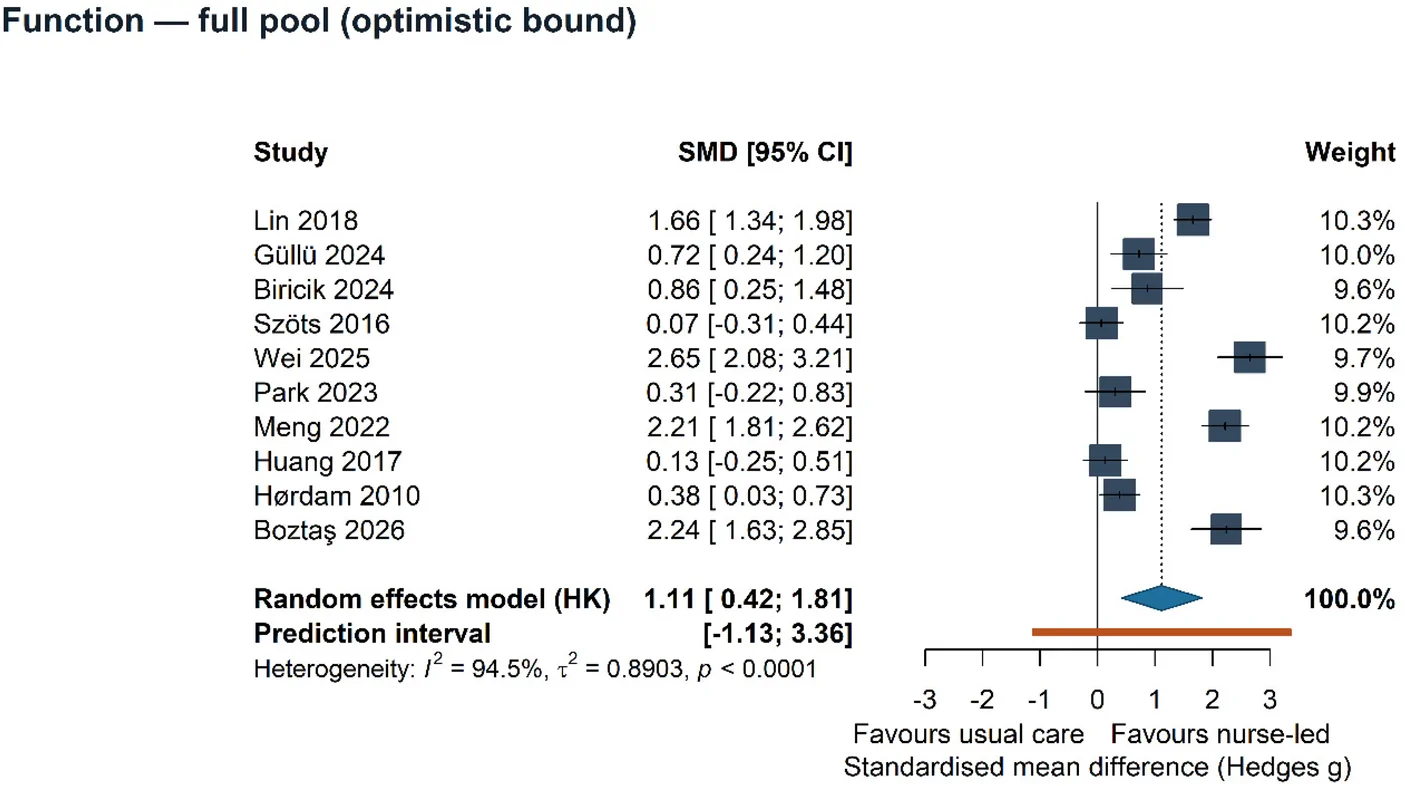
Forest plot of nurse-led rehabilitation versus usual care for functional recovery (10 trials). Hedges *g* with 95% confidence intervals; the diamond is the random-effects pooled estimate (REML with Hartung–Knapp adjustment) and the bar beneath it is the 95% prediction interval.

#### Self-efficacy

Five trials (*n* = 503) contributed to the self-efficacy analysis. The pooled effect was large, but its confidence interval reached the null (*g* = 1.10, 95% CI 0.00–2.20; *I*^2^ = 93.8%, *τ*^2^ = 0.73), and the prediction interval was correspondingly wide (−1.52 to 3.71). The self-efficacy estimate was the least stable of the three primary outcomes: Omitting any of the four larger trials moved the confidence interval to include zero, so the signal should be regarded as suggestive rather than definitive (Table 2, Figure 3).

**Figure 3:**
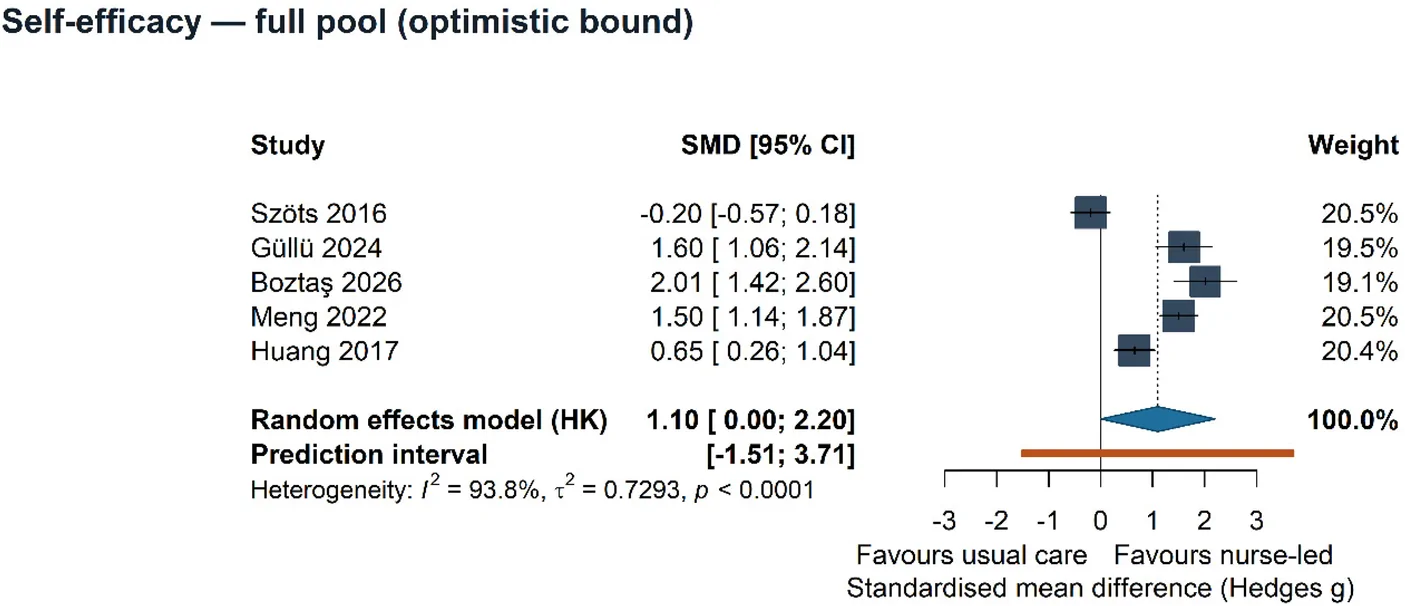
Forest plot for self-efficacy (five trials).

#### Quality of life

Four trials (*n* = 515) reported health-related quality of life. The pooled effect was moderate, and its confidence interval excluded the null (*g* = 0.77, 95% CI 0.08–1.46; *I*^2^ = 81.7%, *τ*^2^ = 0.15), although the prediction interval again spanned zero (−0.65 to 2.20). Quality of life showed the clearest subgroup signal by joint (see subgroup analyses) and was sensitive to the removal of Huang et al. (27), after which heterogeneity fell sharply (see sensitivity and robustness analyses) (Table 2, Figure 4).

**Figure 4:**
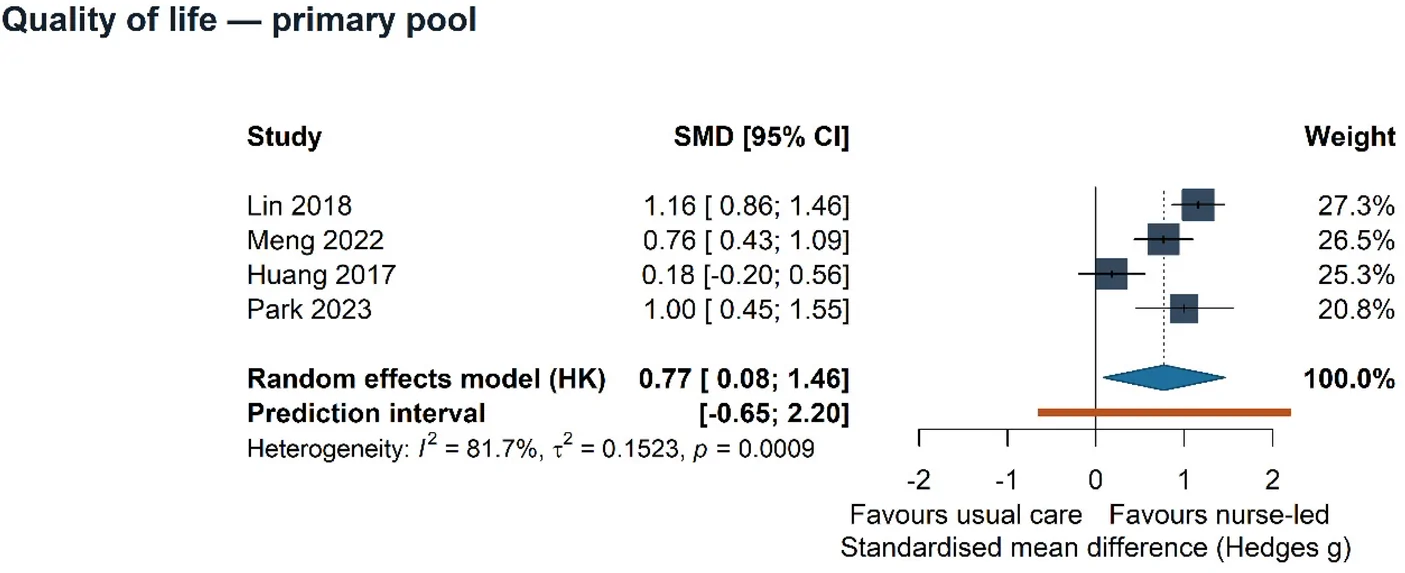
Forest plot for health-related quality of life (four trials).

### Sensitivity and robustness analyses

Three sets of sensitivity analyses were undertaken (full numerical results in Supplementary Table S1; leave-one-out estimates in Supplementary Table S2).

#### Leave-one-out

For functional recovery, the pooled estimate was stable, ranging from *g* = 0.95 [omitting Wei et al. (26)] to *g* = 1.23 [omitting Szöts et al. (20)], with the confidence interval excluding zero in every iteration (Figure 5). For self-efficacy, by contrast, removing any of Güllü and Tosun (21), Boztaş et al. (22), Meng et al. (25), or Huang et al. (27) shifted the confidence interval across the null, confirming the fragility noted earlier. For quality of life, removing Huang et al. (27) reduced *I*^2^ from 82% to 33% and left a precise positive estimate (*g* = 0.98, 95% CI 0.44–1.51), identifying that trial as the principal source of inconsistency.

**Figure 5:**
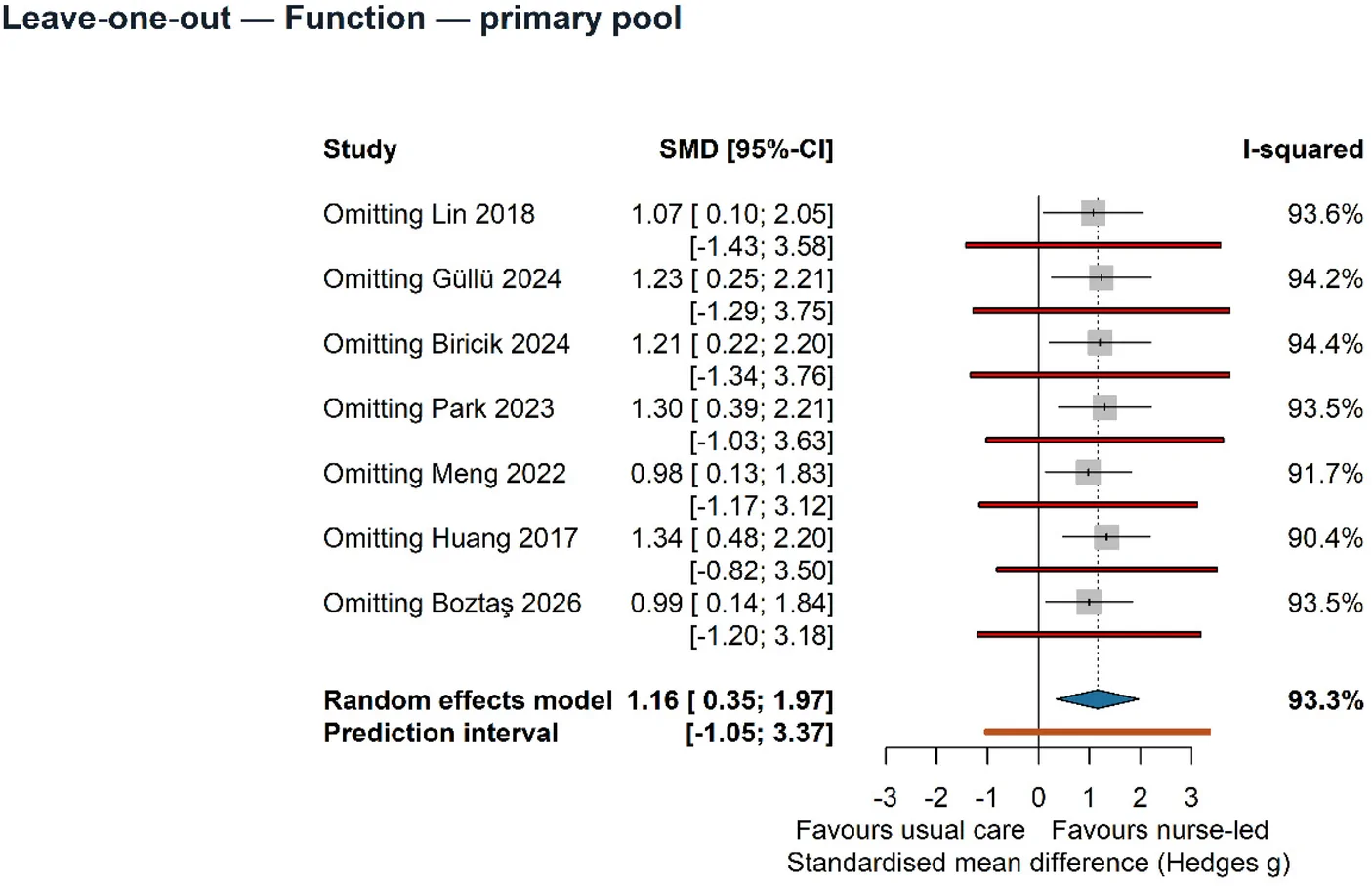
Leave-one-out analysis for functional recovery. Each row shows the pooled estimate after omitting the named trial; the confidence interval excludes the null throughout, demonstrating robustness.

#### Risk-of-bias restriction

Excluding the single high-risk trial (28) did not materially change functional recovery (*g* = 1.20, 95% CI 0.43–1.97; Supplementary Table S1).

#### Measurement restriction

Restricting self-efficacy to validated scales left a similar point estimate but a confidence interval that crossed zero (*g* = 1.22, 95% CI −0.34 to 2.78). Restricting quality of life to standard HRQoL instruments behaved likewise (*g* = 0.71, 95% CI −0.51 to 1.93), reflecting loss of power rather than a change in direction.

### Subgroup analyses

#### By joint (hip vs. knee)

Effects were examined separately for knee and hip replacement (Table 3). For functional recovery, the pooled effect was directionally larger after knee (seven trials; *g* = 1.21, 95% CI 0.30–2.11) than hip surgery (three trials; *g* = 0.91, 95% CI −1.91 to 3.73), but the random-effects test for subgroup differences was non-significant (*χ*^2^ = 0.16, df = 1, *p* = 0.69), and both subgroups remained highly heterogeneous. Self-efficacy was near identical across joints (knee, three trials, *g* = 1.12; hip, two trials, *g* = 1.08; *p* = 0.96). The only material between-joint difference was for quality of life (*p* = 0.032): The knee subgroup showed a consistent, homogeneous benefit (two trials; *g* = 1.12, 95% CI 0.28–1.96; *I*^2^ = 0%), whereas the hip subgroup was small, heterogeneous, and non-significant (two trials; *g* = 0.48, 95% CI −3.23 to 4.19) (Figure 6). Because each subgroup contained only two to seven trials, these comparisons are exploratory and hypothesis-generating.

**Table 3:** Subgroup analysis by joint (hip vs. knee) for the three primary outcomes.

Outcome	Joint	*k*	*g*	95% CI	*I*^2^ (%)	*p* (between)
Functional recovery	Knee	7	1.21	0.30 to 2.11	93.6	0.69
	Hip	3	0.91	−1.91 to 3.73	96.9	
Self-efficacy	Knee	3	1.12	−1.80 to 4.05	96.1	0.96
	Hip	2	1.08	−4.33 to 6.49	89.9	
Quality of life	Knee	2	1.12	0.28 to 1.96	0	0.032
	Hip	2	0.48	−3.23 to 4.19	80.7	

*k*, number of trials; *g*, pooled Hedges *g* (REML + Hartung–Knapp); CI, confidence interval; *I*^2^, heterogeneity. The between-subgroup *p*-value is from the random-effects test for subgroup differences. Wide intervals in the hip and self-efficacy subgroups reflect the small number of trials and the conservative Hartung–Knapp adjustment.

**Figure 6:**
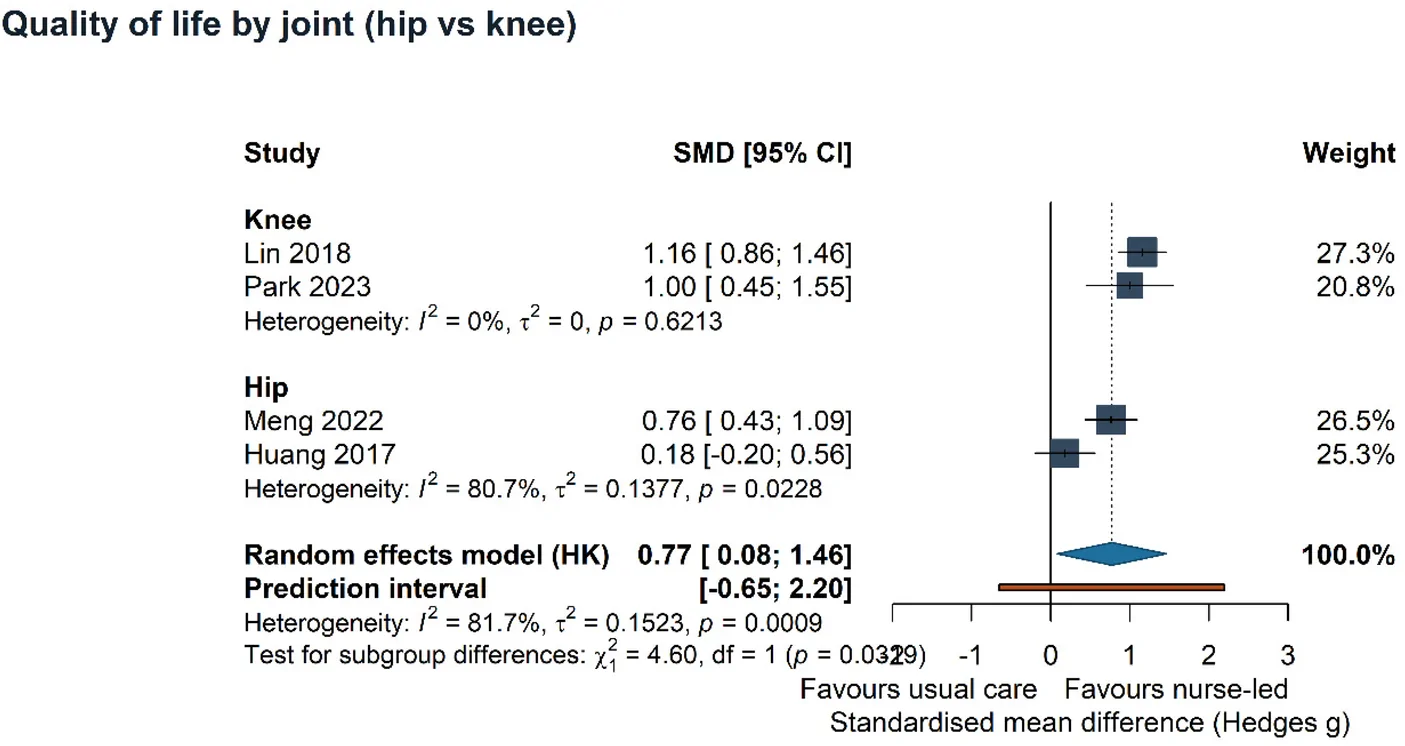
Quality of life by joint (hip vs. knee). The knee subgroup shows a homogeneous benefit (*I*^2^ = 0%); the between-subgroup difference is significant (*p* = 0.032).

#### By intervention delivery model

Functional recovery was further stratified by how the nurse-led program was delivered (Figure 7). The between-subgroup difference was significant on the random-effects test (*χ*^2^ = 10.90, df = 3, *p* = 0.012). Digital interventions yielded the largest point estimate (three trials; *g* = 1.92, 95% CI −0.40 to 4.24), followed by multicomponent (three trials; *g* = 1.09, 95% CI −1.41 to 3.59) and in-person programs (two trials; *g* = 0.90, 95% CI −8.79 to 10.59), while telephone-only delivery showed the smallest effect (two trials; *g* = 0.23, 95% CI −1.77 to 2.24). Notably, the telephone subgroup was the only one with low heterogeneity (*I*^2^ = 30.6%), whereas the other modalities remained highly heterogeneous (*I*^2^ = 89%–97%). All subgroup confidence intervals crossed the null. The apparent gradient should be interpreted cautiously: Three of the four digital trials were also the principal contributors to overall heterogeneity, so the difference may reflect which trials adopted digital delivery rather than a true effect of modality.

**Figure 7:**
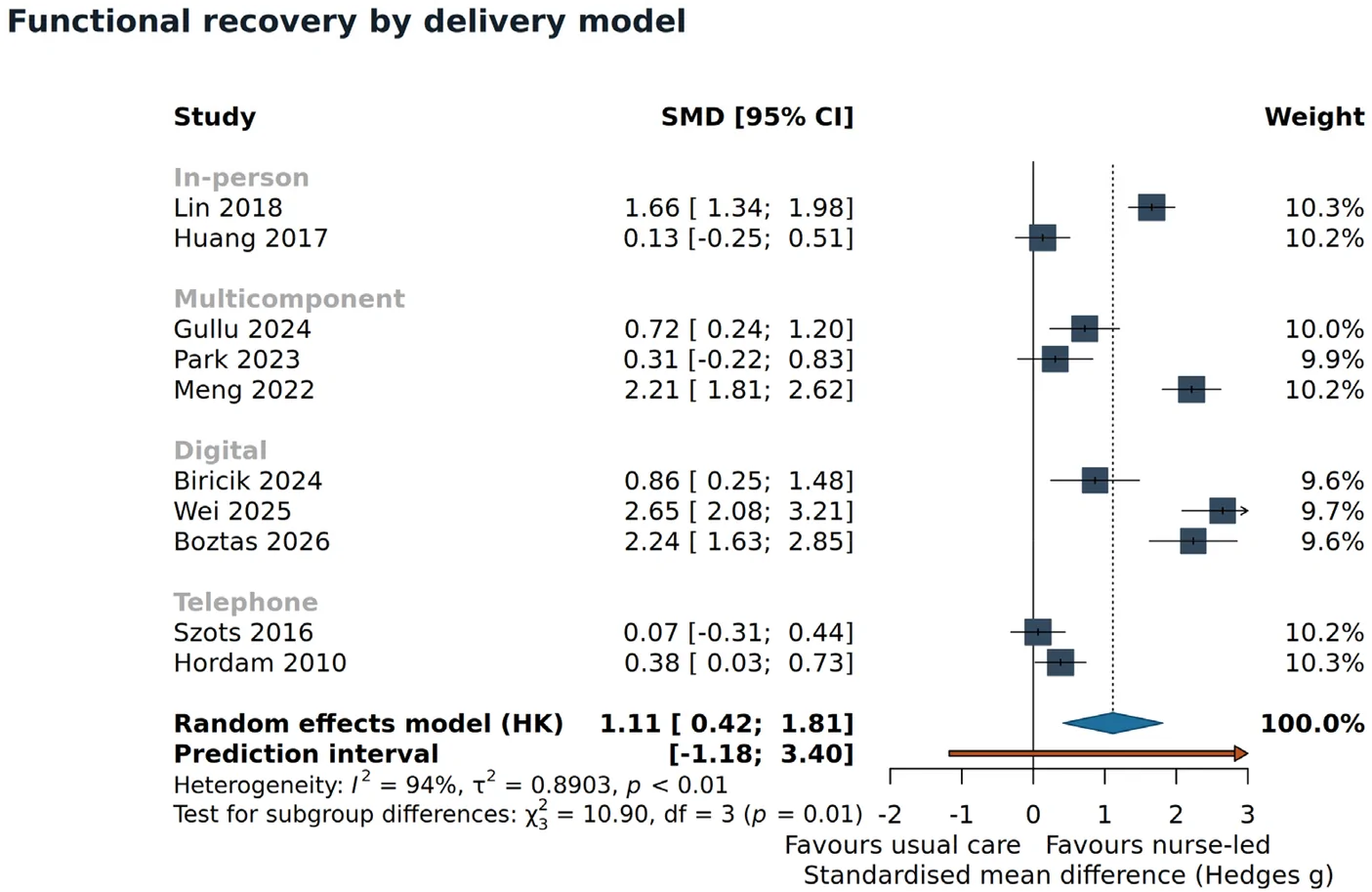
Functional recovery, with trials grouped by nurse-led delivery model. Only the overall pooled estimate (diamond; REML + Hartung–Knapp) and its 95% prediction interval are shown; per-subgroup diamonds are omitted because each modality contained only two to three trials, producing unstable, very wide intervals. The between-subgroup test is reported in the footer (*χ*^2^ = 10.90, df = 3, *p* = 0.012). The *x*-axis is truncated at −2 and 3; intervals beyond these limits are shown with arrowheads.

### Trial reported with adjusted estimates

Wang et al. (24) (*n* = 86; mixed hip and knee) reported covariate-adjusted between-group differences, and so that study was not entered into the pooled standardized effects. Pooling was not possible for two reasons: Arm-level means and standard deviations were not reported, so a standardized mean difference (Hedges *g*) could not be computed; and covariate-adjusted differences estimate a systematically different quantity from unadjusted postintervention contrasts, so combining the two in one pooled effect would mix incompatible estimands. Narrative synthesis was therefore the prespecified approach for trials reporting in this format (see Methods). Its results were directionally consistent with the meta-analysis across every domain: Self-efficacy favored nurse-led care (adjusted MD 0.72, 95% CI 0.31–1.14; *p* < 0.001), as did physical function (adjusted MD 4.57 on HOOS/KOOS-PS, 95% CI 1.24–7.90; *p* = 0.007) and health-related quality of life (EQ-5D adjusted MD 0.06, 95% CI 0.01–0.10; *p* = 0.018). Anxiety and depression were lower in the intervention arm (HADS-A −0.51, 95% CI −0.91 to −0.10; HADS-D −0.37, 95% CI −0.66 to −0.08). Full adjusted estimates are provided in Supplementary Table S3.

### Secondary outcomes

Three secondary outcomes were pooled from two to three trials each (Table 2). Anxiety/depression favored nurse-led care on the point estimate (*g* = 0.78), but the confidence interval crossed zero (−0.17 to 1.73). Exercise adherence showed a very large but imprecise effect (*g* = 2.26, 95% CI −0.36 to 4.87) with low heterogeneity (*I*^2^ = 34%); the width of the interval reflects the small-sample Hartung–Knapp adjustment with only two trials. Pain was essentially indeterminate (*g* = 0.35, 95% CI −0.91 to 1.60). These outcomes should be interpreted as insufficient evidence rather than evidence of no effect. Corresponding forest plots appear in Supplementary Figures S3–S5.

### Heterogeneity and small-study effects

Heterogeneity was high for all three primary outcomes and was explored through influence diagnostics. Baujat analysis identified the high-effect trials [notably Meng et al. (25) and Boztaş et al. (22)] as the joint contributors to heterogeneity and to the pooled estimate, mirroring the leave-one-out results. For functional recovery, the only pool with ≥10 trials, a contour-enhanced funnel plot, showed visible asymmetry, with the smaller trials lying on the side favoring nurse-led care. The cumulative analysis by year showed the estimate consolidating into a positive, significant effect as the later high-effect trials accrued (Supplementary Figure S9). Small-study effects therefore cannot be excluded for functional recovery, and the pooled estimate should be read as a likely upper bound.

### Certainty of evidence (grade)

Certainty was rated low for all three primary outcomes (Table 4). Each was downgraded for risk of bias (predominantly unblinded patient-reported outcomes, with one high-risk trial), inconsistency (*I*^2^ ≥ 82%), and imprecision (few small trials with prediction intervals crossing the null). Indirectness was a minor concern given the mixed-cohort and varied delivery models. Publication bias was formally assessed only for functional recovery (*k* = 10) and was not detected on Egger's test, though the funnel asymmetry noted earlier tempers this. The overall picture is of a consistent direction of benefit across outcomes set against low certainty in the magnitude of effect.

**Table 4:** Grade summary of findings for the three primary outcomes.

Outcome	*k*	*n*	*g* (95% CI)	Risk of bias	Inconsistency	Imprecision	Certainty
Functional recovery	10	1,021	1.11 (0.42, 1.81)	Serious	Serious (*I*^2^ = 94%)	Serious	⊕⊕◯◯ Low
Self-efficacy	5	503	1.10 (0.00, 2.20)	Serious	Serious (*I*^2^ = 94%)	Serious	⊕⊕◯◯ Low
Quality of life	4	515	0.77 (0.08, 1.46)	Serious	Serious (*I*^2^ = 82%)	Serious	⊕⊕◯◯ Low

Certainty downgraded from an initial “high” (RCT evidence); ⊕⊕◯◯ Low. Indirectness rated “some” for all outcomes (mixed cohort, varied delivery).

### Summary of findings

Nurse-led rehabilitation after hip or knee arthroplasty was associated with a large improvement in physical function, a large but less certain improvement in self-efficacy, and a moderate improvement in quality of life, with secondary outcomes pointing in the same direction. The functional recovery effect was robust to leave-one-out and to exclusion of the high-risk trial; the quality-of-life benefit was clearest and most homogeneous after knee replacement; and the self-efficacy effect, while large, was statistically fragile. All estimates were highly heterogeneous, every prediction interval crossed the null, and small-study effects could not be excluded for functional recovery, so the evidence consistently favors nurse-led care in direction but remains of low certainty in magnitude.

## Discussion

This systematic review and meta-analysis of 11 randomized controlled trials (1,107 participants) found that nurse-led rehabilitation after total hip or knee arthroplasty was associated with a large improvement in physical function (Hedges *g* = 1.11, 95% CI 0.42–1.81), a large but less certain improvement in self-efficacy (*g* = 1.10, 95% CI 0.00–2.20), and a moderate improvement in health-related quality of life (*g* = 0.77, 95% CI 0.08–1.46). The direction of effect favored nurse-led care across every primary and secondary outcome, and the only trial reporting solely adjusted estimates was concordant on all domains (24). However, heterogeneity was substantial for all three primary outcomes, every prediction interval crossed the null, and the GRADE certainty of evidence was low throughout. The central message is therefore one of a consistent and clinically meaningful direction of benefit set against genuine uncertainty about its magnitude.

The magnitude of these effects, and not only their statistical significance, warrants emphasis. By conventional benchmarks, a standardized mean difference of about 0.5 is moderate and one of about 0.8 is large, so the pooled estimates for function (*g* = 1.11) and self-efficacy (*g* = 1.10) represent large effects, and that for quality of life (*g* = 0.77) represents a moderate-to-large effect. Translated clinically, an effect of this order implies that a substantial majority of patients receiving nurse-led rehabilitation would report better function than the average patient receiving usual care, a difference most patients and clinicians would notice in practice. This interpretation must nonetheless be tempered on two counts: Standardized effects are liable to inflation when outcomes are unblinded and patient-reported, as they were here, and the wide prediction intervals mean the benefit actually experienced could be considerably smaller in any individual setting. Because the included trials used heterogeneous instruments, we could not anchor these effects to instrument-specific minimal clinically important differences; doing so should be a priority for future trials, so that benefit can be expressed in units that clinicians recognize rather than as a standardized score. The clinical importance of nurse-led rehabilitation therefore rests on the consistent direction and generally moderate-to-large size of the effect across every outcome, rather than on statistical significance alone.

### Interpretation in the context of existing evidence

These findings extend, rather than contradict, the existing literature. Because most of the postoperative recovery trajectory now unfolds at home under enhanced recovery pathways, the postdischarge period is the decisive window for rehabilitation, and nurses increasingly occupy its center (4–6). Our results provide quantitative support for that role: Structured, nurse-delivered education and self-management were associated with better patient-reported recovery than usual care.

A discharge readiness framework helps to organize these observations and to clarify why a nurse-led model should matter. Readiness for hospital discharge, understood as a patient's physical, psychological, and knowledge-based preparedness to manage recovery at home, is a nurse-sensitive outcome that lies directly on the causal path between perioperative care and postdischarge function. Because enhanced recovery pathways discharge patients earlier and transfer responsibility for rehabilitation to them sooner, the quality of nurse-led preparation at the point of discharge plausibly determines how faithfully home-exercise and self-management programs are subsequently carried out. Within this framework, the interventions synthesized here act by raising discharge readiness and early self-management capability, which in turn support adherence and self-efficacy and, ultimately, functional recovery and quality of life. This chain—nurse-led education and support, leading to discharge readiness, then to self-efficacy and adherence, and finally to functional recovery—offers a coherent conceptual rationale for the pattern of effects we observed, in which the largest pooled effects were seen for the proximal target (self-efficacy) and for function. It also identifies discharge readiness as a measurable intermediate endpoint that future trials could use to test the mechanism directly.

The self-efficacy signal is mechanistically coherent. Self-efficacy is an established, modifiable predictor of functional outcome after arthroplasty (9), and several of the included interventions were explicitly built on self-efficacy or empowerment frameworks (22, 25, 27). That the largest pooled effect in our review was for self-efficacy itself—even if statistically fragile—suggests that these interventions acted on their intended target, with functional and quality-of-life gains plausibly following from improved confidence and adherence. This aligns with evidence that home-exercise adherence is strongly shaped by guidance, feedback, and motivational support (8). The broader literature on educator-led chronic disease management is consistent with this mechanism: Structured education delivered by clinicians has been associated with improved medication adherence and quality of life in other conditions, including type 2 diabetes, although such evidence often derives from uncontrolled before–after studies and must be extrapolated to the surgical setting with caution (29).

Our delivery model analysis is consistent with the growing telerehabilitation literature, which reports that technology-assisted and mobile application programs can improve pain, function, and adherence after lower-limb arthroplasty and in knee osteoarthritis more broadly (11–13). According to our data, digitally delivered interventions produced the largest functional effect and telephone-only delivery produced the smallest, with a significant between-subgroup difference. We caution strongly against over-interpreting this gradient; however, three of the four digital trials (22, 23, 26) were also the principal contributors to overall heterogeneity, so the apparent superiority of digital delivery may reflect which trials happened to adopt it rather than a true effect of modality. Conceptual frameworks for nurse-led postsurgical care continue to emphasize structured, empowering, multicomponent support regardless of delivery channel (10).

Variation in the components of the nurse-led programs is itself a likely source of the differing effects and merits explicit consideration. Although all interventions shared a nurse-led core, they combined different active ingredients in different proportions: structured patient education, guided or progressive exercise instruction, explicit self-efficacy or empowerment scaffolding, remote symptom monitoring, and scheduled follow-up contacts of varying number and duration. Programs built explicitly on self-efficacy or empowerment frameworks, and those that paired education with active exercise guidance, tended to report the largest effects, whereas single-channel telephone follow-up, which chiefly offered reassurance and problem-solving rather than structured exercise instruction, produced the smallest effects. The delivery model subgroup difference should therefore be read partly as a difference in intervention content rather than in channel alone, since the digital and multicomponent programs generally delivered a richer bundle of active components than telephone-only contact. This confounding of channel with content, together with the small number of trials per modality and the clustering of high-effect trials within the digital subgroup, is a further reason to treat the apparent superiority of any single delivery format as hypothesis-generating rather than definitive, and it reinforces the case for trials that report intervention content in sufficient detail to permit component-level synthesis.

### Heterogeneity and robustness

A defining feature of this evidence base is its heterogeneity (*I*^2^ 82%–95% for the primary outcomes). We addressed this through prediction intervals, influence diagnostics, and extensive sensitivity analyses rather than relying on point estimates alone. The functional recovery effect proved robust: Leave-one-out analysis left the confidence interval above the null in every iteration, and excluding the single high-risk trial (28) did not materially change the estimate. The self-efficacy effect was more fragile—omitting any of the larger trials (21, 22, 25, 27) moved the interval across the null—so it should be regarded as suggestive. For quality of life, a single trial (27) accounted for most of the inconsistency: Its removal reduced *I*^2^ from 82% to 33% while leaving a precise positive estimate. The subgroup analyses by joint were under-powered (two to seven trials per group) and should be read as exploratory; only the quality-of-life difference between knee and hip reached significance, and the wide Hartung–Knapp intervals in the small subgroups underline how little can be concluded from them. Importantly, for functional recovery—the only outcome with at least 10 trials—a contour-enhanced funnel plot showed asymmetry consistent with small-study effects, so the pooled estimate is best interpreted as a likely upper bound on the true benefit.

It is important to distinguish statistical from clinical heterogeneity when interpreting these pooled estimates. The high *I*^2^ values reflect genuine clinical diversity across the trials rather than a statistical artifact: The trials differed in the joint operated (hip vs. knee), in the content and intensity of the nurse-led program, in the nature of the “usual care” comparator (which ranged from brief discharge advice to structured routine physiotherapy, so that the same intervention would generate a larger effect where the comparator was weaker), in the outcome instruments used, and in the timing of assessment. Cross-national differences in healthcare organization are a further plausible source: The trials spanned health systems in East Asia, Türkiye, and Northern Europe that differ in nursing autonomy and scope of practice, in the structure and intensity of routine postoperative rehabilitation, and in the maturity of community-based and digital follow-up, so that nominally similar nurse-led programs were delivered, and compared against different versions of usual care, in organizationally distinct contexts. A practical consequence is that the random-effects pooled effect should be read as an average across a heterogeneous set of implementations rather than as a single transportable value: With *I*^2^ between 82% and 95%, the mean effect need not correspond to the effect expected in any particular clinic. This is precisely why we report a prediction interval alongside every pooled estimate, and because each prediction interval crossed the null, the honest summary is that nurse-led rehabilitation is very likely to help on average while its effect in a specific future setting could plausibly range from negligible to large. The pooled point estimates are therefore best used to establish the direction and general plausibility of benefit, whereas decisions for a given service should weigh local case mix, the strength of existing usual care, and the intended program components against this residual uncertainty.

### Strengths and limitations

This review has several strengths. It was conducted and reported according to PRISMA 2020 (14), used the Cochrane RoB 2 tool (16) and GRADE (17) for transparent appraisal, and applied a conservative analytic strategy—random-effects pooling with the Hartung–Knapp adjustment, prediction intervals, and prespecified sensitivity and subgroup analyses in accordance with the Cochrane Handbook (15)—that guards against overstating precision when trials are few and small. The narrative integration of trial that reported only adjusted estimates (24), rather than its exclusion, allowed all eligible evidence to inform the conclusions.

The limitations largely reflect the primary evidence. First, no trial was at low overall risk of bias, and the pervasive limitation was outcome measurement: The interventions could not be blinded, and the outcomes were patient-reported, so detection bias cannot be excluded and is likely to inflate effect sizes. Second, statistical heterogeneity was high, and the wide prediction intervals mean the effect in any individual future setting remains uncertain. Third, the trials were clinically diverse spanning different countries, instruments, and delivery formats (18–28), and several standard deviations were reconstructed from reported statistics, a recognized source of imprecision. Fourth, the included trials were modest in size, and the secondary outcomes were informed by only two to three trials each, so those estimates are insufficient rather than null. Fifth, the review was restricted to English-language reports, introducing possible language bias the potential impact of which deserves emphasis: Given that the included trials originated predominantly in East Asia, Türkiye, and Northern Europe, eligible trials published in Chinese, Turkish, Korean, or other languages plausibly exist, and their omission could shift the pooled estimates in either direction. Funnel-plot asymmetry suggests small-study effects for the best-evidenced outcome. Sixth, the electronic search covered PubMed, Scopus, Web of Science, and ClinicalTrials.gov and did not include the nursing-specific databases CINAHL and Embase. We combined three large multidisciplinary databases with a trial registry and hand-searched the reference lists of all included studies and of relevant systematic reviews; this hand-searching did not identify any additional eligible randomized trials beyond those already retrieved, which offers some reassurance as to the completeness of the evidence located. Nevertheless, trials indexed exclusively in CINAHL or Embase may have been missed. Because these databases index a number of European and Asian journals that are not covered by MEDLINE, and the included trials were conducted predominantly in East Asia, Türkiye, and Northern Europe, we regard this as a material rather than a nominal limitation, and it should be weighed alongside the English-language restriction when judging the completeness of the evidence base. Any trials missed on this account would most plausibly be small; given the funnel-plot asymmetry already observed for functional recovery, their inclusion would be more likely to attenuate rather than increase the pooled estimate, which would reinforce rather than overturn our interpretation of that estimate as a likely upper bound. Future updates of this review should incorporate CINAHL and Embase, together with the Cochrane Central Register of Controlled Trials (CENTRAL), which is the most comprehensive single source of reports of randomized trials. Evidence concerning nurses' adherence to clinical safety processes is included as contextual support for the broader determinants of nursing process fidelity rather than as direct evidence on patient rehabilitation (7).

### Implications for practice and research

For clinical practice, the consistent direction of benefit across function, self-efficacy, and quality of life supports the continued integration of nurse-led rehabilitation into postarthroplasty care pathways, particularly given that nurses already occupy this role within enhanced recovery programs (6) and that the interventions are low risk and scalable. The low certainty of evidence, however, means that these programs should be implemented as promising rather than proven, ideally with local evaluation.

Several concrete recommendations follow for nursing practice, discharge planning, and postoperative rehabilitation. First, nurse-led rehabilitation is best embedded within existing enhanced recovery pathways rather than added as a stand-alone service, with nurses formally responsible for preoperative education, a structured assessment of discharge readiness, and scheduled postdischarge follow-up. Second, discharge planning should pair written and demonstrated home-exercise instruction with explicit self-efficacy components—collaborative goal-setting, performance feedback, and problem-solving for anticipated barriers—because confidence to perform recovery behaviors, rather than information alone, appears to drive adherence. Third, at least one proactive nurse-initiated contact during the early postdischarge weeks, delivered by telephone or digital platform according to local resources and patient digital access, allows early detection of pain, wound, or mobility problems and timely reinforcement of the exercise program; the choice of channel should be guided by feasibility and equity of access rather than by the apparent superiority of digital delivery in our subgroup analysis, which was confounded and imprecise. Fourth, because the certainty of evidence is low, services adopting these programs should audit patient-reported function, self-efficacy, and quality of life locally, so that implementation simultaneously serves as evaluation and contributes to the evidence base. These recommendations are deliberately low cost and scalable and are compatible with the coordinating role that nurses already hold within perioperative care.

These recommendations should be situated within multidisciplinary rehabilitation pathways rather than read as advocating for nurse-led care in isolation. In the included trials, nurse-led programs operated alongside, not instead of, surgical follow-up and routine physiotherapy, and the most plausible model of benefit is complementary: Physiotherapists prescribe and progress exercise, while nurses provide the education, monitoring, and self-efficacy support that sustain adherence between formal contacts, within teams that may also include rehabilitation physicians and occupational therapists. The organizational implications will differ across health systems. Where community nursing and digital infrastructure are well developed, nurse-led follow-up can extend continuity of care into the home at modest cost; where nursing workforces are constrained, task-sharing arrangements and the regulated scope of nursing practice will determine what is feasible, and future implementation studies should specify these organizational parameters explicitly. For health policy and workforce planning, the findings support investment in postdischarge nursing capacity within enhanced recovery pathways, including training in self-management support and remote follow-up, and they are particularly relevant to systems undergoing reforms toward community-based, integrated, and digitally enabled models of care in which health professionals are assuming expanded clinical and organizational roles (30, 31).

For research, the priority is clear: Adequately powered randomized trials with blinded or objectively measured outcomes, standardized self-efficacy and function instruments, and preregistered protocols. Future trials should report sufficient data for pooling, directly compare delivery modalities (digital vs. telephone vs. in-person) head to head rather than across trials, and examine whether self-efficacy mediates downstream functional and quality-of-life gains. Until such trials exist, the magnitude of benefit from nurse-led rehabilitation will remain uncertain even as its direction appears dependable.

## Conclusion

Nurse-led rehabilitation after total hip or knee arthroplasty was associated with improved physical function, self-efficacy, and quality of life compared with usual care, with all outcomes pointing in the same favorable direction. The functional recovery benefit was the most robust, withstanding leave-one-out and risk-of-bias sensitivity analyses, whereas the self-efficacy effect, though large, was statistically fragile, and the quality-of-life benefit was clearest after knee replacement. These findings are tempered by substantial heterogeneity, prediction intervals that crossed the null, possible small-study effects, and an overall low certainty of evidence driven largely by unblinded patient-reported outcomes. Funnel-plot asymmetry for functional recovery raises the further possibility of small-study or publication bias inflating the pooled estimate, which should therefore be regarded as a likely upper bound on the true benefit. Nurse-led rehabilitation is therefore a promising, low-risk, and scalable adjunct to postarthroplasty recovery that can reasonably be incorporated into existing care pathways as one component of multidisciplinary rehabilitation, complementing rather than replacing physiotherapy-led care. Adequately powered trials with blinded outcome assessment are needed to confirm the size of its benefit. Beyond larger trials, future research should identify the specific intervention components responsible for the observed benefits, evaluate cost-effectiveness, and determine how nurse-led rehabilitation is best integrated within multidisciplinary models of care.

## Data Availability

The original contributions presented in the study are included in the article/Supplementary Material, further inquiries can be directed to the corresponding author.
