# Supplementary material for: Nurse-led rehabilitation after total hip or knee arthroplasty: a systematic review and meta-analysis of functional recovery, self-efficacy, and quality of life
Source: Front Med (Lausanne). 2026 Oct 5;13:1911533. doi: 10.3389/fmed.2026.1911533 (PMC13677350; doi:10.3389/fmed.2026.1911533)
Supplement: Supplementary file 1 [file Datasheet1.docx]

**Supplementary Results**

**Supplementary Figure S1. Risk-of-bias summary (RoB 2), weighted by study size, across the five domains.**

**Supplementary Figure S2. Study-level RoB 2 traffic-light plot. D1 randomisation; D2 deviations from intended intervention; D3 missing outcome data; D4 outcome measurement; D5 selection of the reported result.**

## B. Secondary-outcome forest plots

**Supplementary Figure S3. Anxiety/depression (psychological distress), 3 trials.**

**Supplementary Figure S4. Pain, 3 trials (trials reporting usable standard deviations).**

**Supplementary Figure S5. adherence, 2 trials. The wide interval reflects the small-sample Hartung–Knapp adjustment with k = 2.**

## Subgroup analyses

The quality-of-life-by-joint and delivery-model subgroup analyses appear in the main text (Figures 6 and 7). The two subgroup forest plots below complete the by-joint analysis for the remaining primary outcomes. For consistency with the main-text figures, per-subgroup pooled diamonds are omitted (each subgroup contained only two to seven trials, producing unstable, very wide intervals); studies remain grouped with per-subgroup heterogeneity, and the between-subgroup difference test is reported in each footer.

**Supplementary Figure S6. Functional recovery by joint (hip vs knee). Between-subgroup difference χ² = 0.16, df = 1, p = 0.69. Per-subgroup diamonds omitted; only the overall pooled estimate is shown.**

**Supplementary Figure S7. *Self-efficacy by joint (hip vs knee). Between-subgroup difference χ² = 0.00, df = 1, p = 0.96. Per-subgroup diamonds omitted; only the overall pooled estimate is shown.***

**Supplementary Figure S8. *Contour-enhanced funnel plot for functional recovery (10 trials). Shaded bands mark conventional significance contours; visible asymmetry suggests possible small-study effects.***

**Supplementary Figure S9. *Cumulative meta-analysis by year for functional recovery, showing consolidation of the estimate as later high-effect trials accrued.***

## Supplementary tables

**Supplementary Table S1. Sensitivity analyses for the three primary outcomes (main pool vs risk-of-bias and measurement restrictions).**

| **Analysis** | **k** | **g** | **95% CI** | **95% PI** | **I² (%)** | **τ²** |
| --- | --- | --- | --- | --- | --- | --- |
| Function — main | 10 | 1.11 | 0.42 to 1.81 | −1.13 to 3.36 | 94.5 | 0.890 |
| Function — excl. high RoB (Hørdam) | 9 | 1.20 | 0.43 to 1.97 | −1.16 to 3.56 | 94.6 | 0.935 |
| Self-efficacy — main | 5 | 1.10 | 0.00 to 2.20 | −1.52 to 3.71 | 93.8 | 0.729 |
| Self-efficacy — validated scales only | 4 | 1.22 | −0.34 to 2.78 | −2.20 to 4.63 | 95.2 | 0.911 |
| QoL — main | 4 | 0.77 | 0.08 to 1.46 | −0.65 to 2.20 | 81.7 | 0.152 |
| QoL — standard HRQoL only | 3 | 0.71 | −0.51 to 1.93 | −1.61 to 3.02 | 87.3 | 0.210 |

***Abbreviations / notes:*** *PI, prediction interval. Restrictions reduce k and widen intervals chiefly through loss of power.*

**Supplementary Table S2. Leave-one-out estimates for the three primary outcomes.**

| **Outcome** | **Omitted trial** | **g** | **95% CI** | **I² (%)** |
| --- | --- | --- | --- | --- |
| Functional recovery | Omitting Lin 2018 | 1.05 | 0.27 to 1.83 | 94.4 |
|  | Omitting Güllü 2024 | 1.16 | 0.37 to 1.95 | 95.0 |
|  | Omitting Biricik 2024 | 1.14 | 0.35 to 1.93 | 95.1 |
|  | Omitting Szöts 2016 | 1.23 | 0.50 to 1.97 | 94.1 |
|  | Omitting Wei 2025 | 0.95 | 0.28 to 1.61 | 93.8 |
|  | Omitting Park 2023 | 1.20 | 0.44 to 1.96 | 94.8 |
|  | Omitting Meng 2022 | 0.99 | 0.26 to 1.72 | 93.6 |
|  | Omitting Huang 2017 | 1.23 | 0.48 to 1.97 | 94.2 |
|  | Omitting Hørdam 2010 | 1.20 | 0.43 to 1.97 | 94.6 |
|  | Omitting Boztaş 2026 | 0.99 | 0.27 to 1.72 | 94.5 |
| Self-efficacy | Omitting Szöts 2016 | 1.42 | 0.51 to 2.33 | 84.1 |
|  | Omitting Güllü 2024 | 0.98 | −0.56 to 2.52 | 94.8 |
|  | Omitting Boztaş 2026 | 0.88 | −0.46 to 2.22 | 94.0 |
|  | Omitting Meng 2022 | 1.00 | −0.58 to 2.57 | 94.2 |
|  | Omitting Huang 2017 | 1.22 | −0.34 to 2.78 | 95.2 |
| Quality of life | Omitting Lin 2018 | 0.62 | −0.41 to 1.66 | 74.1 |
|  | Omitting Meng 2022 | 0.78 | −0.55 to 2.10 | 87.7 |
|  | Omitting Huang 2017 | 0.98 | 0.44 to 1.51 | 33.0 |
|  | Omitting Park 2023 | 0.71 | −0.51 to 1.93 | 87.3 |

***Abbreviations / notes:*** *Each row omits one trial and re-pools the remainder. For functional recovery the 95% CI excludes zero throughout; for self-efficacy several omissions move the CI across the null; for quality of life, omitting Huang 2017 collapses heterogeneity (I² = 33%).*

**Supplementary Table S3. Covariate-adjusted between-group differences reported by Wang Q 2023 (analysed narratively, not pooled).**

| **Outcome** | **Adjusted MD** | **95% CI** | **p** |
| --- | --- | --- | --- |
| Self-efficacy (SER) | 0.72 | 0.31 to 1.14 | <0.001 |
| Physical function (HOOS/KOOS-PS) | 4.57 | 1.24 to 7.90 | 0.007 |
| HRQoL (EQ-5D) | 0.06 | 0.01 to 0.10 | 0.018 |
| Anxiety (HADS-A) | −0.51 | −0.91 to −0.10 | 0.015 |
| Depression (HADS-D) | −0.37 | −0.66 to −0.08 | 0.012 |

***Abbreviations / notes:*** *MD, mean difference; SER, self-efficacy for rehabilitation; HOOS/KOOS-PS, Hip/Knee injury and Osteoarthritis Outcome Score – Physical Short-form; EQ-5D, EuroQol-5D; HADS, Hospital Anxiety and Depression Scale. Negative HADS values favour nurse-led care. The subgroup-by-joint summary table is presented in the main text (Table 3).*

**Supplementary Table S4. Core components of the nurse-led interventions in the included trials.**

| **Study** | **Delivery model** | **Structured education** | **Exercise instruction / training** | **Named behavioural framework** | **Digital platform / telemonitoring** | **Telephone follow-up** |
| --- | --- | --- | --- | --- | --- | --- |
| Park & Jeong 2023 | Multicomponent | Yes | Yes | — | — | — |
| Lin 2018 | In-person | Yes | Yes (lower-extremity strength training) | — | — | — |
| Szöts 2016 | Telephone | Yes (telephone-delivered) | — | — | — | Yes |
| Güllü & Tosun 2024 | Multicomponent | Yes | Yes | Transitional Care Model | — | Yes (transitional follow-up) |
| Boztaş 2026 | Digital | Yes (web-based) | — | Self-care agency / self-efficacy | Yes | — |
| Biricik 2024 | Digital | Yes (video-assisted) | — | — | Yes | — |
| Wang Q 2023 | Digital | Yes (app-based) | Yes (app-guided exercises) | — | Yes | — |
| Meng 2022 | Multicomponent | Yes | Yes | Self-efficacy theory (Bandura) | — | — |
| Wei 2025 | Digital | Yes | Yes | Information–Motivation–Behavioural skills (IMB) model | Yes | — |
| Huang 2017 | In-person | Yes | — | Empowerment education | — | — |
| Hørdam 2010 | Telephone | Yes (telephone counselling) | — | — | — | Yes |

*Abbreviations / notes: Components were compiled from the published trial reports and the study characteristics summarized in Table 1 of the main text. "—" indicates that the component was not described as a stated element of the intervention. All interventions were designed and primarily delivered by nurses; usual-care comparators are not shown.*

**Supplementary Methods. Search strategy**

The search combined three concept blocks — arthroplasty, nurse-led care, and rehabilitation/recovery — using controlled vocabulary where available and free-text terms in all databases, restricted to 2010–2026 and English language. The representative PubMed strategy was:

(arthroplasty[MeSH] OR arthroplasty[tiab] OR "joint replacement"[tiab] OR "hip replacement"[tiab] OR "knee replacement"[tiab] OR "hip arthroplasty"[tiab] OR "knee arthroplasty"[tiab]) AND ("nurse-led"[tiab] OR "nurse led"[tiab] OR "nursing intervention"[tiab] OR "nurse navigator"[tiab] OR "rehabilitation nursing"[tiab] OR nurses[MeSH] OR "nurse’s role"[MeSH]) AND (rehabilitation[MeSH] OR rehabilitation[tiab] OR "self-management"[tiab] OR "self management"[tiab] OR education[tiab] OR "patient education"[tiab] OR telerehabilitation[tiab] OR recovery[tiab]) AND ("2010/01/01"[dp] : "2026/12/31"[dp]) AND english[la]

The same three blocks were translated for Scopus (TITLE-ABS-KEY), Web of Science (TS=), and ClinicalTrials.gov (condition: hip OR knee arthroplasty; other terms: nurse-led rehabilitation), with syntax adapted to each interface. Reference lists of included studies and relevant systematic reviews were hand-searched.
